# Demographic and cultural correlates of traditional eating among Alaska Native adults at risk for cardiovascular disease

**DOI:** 10.1371/journal.pone.0275445

**Published:** 2022-09-30

**Authors:** Mark A. Sanders, Marily Oppezzo, Jordan Skan, Neal L. Benowitz, Matthew Schnellbaecher, Judith J. Prochaska

**Affiliations:** 1 Kaiser Permanente Bernard J. Tyson School of Medicine, Pasadena, California, United States of America; 2 Department of Medicine, Stanford Prevention Research Center, Stanford University, Stanford, California, United States of America; 3 Cardiology Department, Alaska Native Tribal Health Consortium, Anchorage, Alaska, United States of America; 4 Department of Medicine, Division of Cardiology, Program in Clinical Pharmacology, and the Center for Tobacco Control Research and Education, UCSF, San Francisco, California, United States of America; PLOS: Public Library of Science, UNITED KINGDOM

## Abstract

This cross-sectional study assessed how traditional eating relates to cultural and community factors. Alaska Native adults from the Norton Sound region were recruited and surveyed between 2015–2018 for a randomized clinical trial of multiple risk behavior change interventions for cardiovascular disease prevention. Participants (n = 291) were 49% female with a mean age of 47 years (SD = 14). A 34-item food frequency questionnaire assessed consumption of foods traditional and nontraditional to the regional Alaska Native diet. A novel measure, termed the “traditional foods index”, was computed as weekly servings of culturally traditional food consumption divided by total foods reported. Overall, the sample’s traditional foods index averaged 21%±16%, with higher values reported by participants assessed in summer (23%±17%) than winter (19%±15%, p<0.05); by women (22%±16%) than men (19%±16%, p < .05); and by residents of smaller communities (22%±17%) than the comparatively larger community of Nome (17%±14%, p<0.05). The traditional foods index was correlated with age (r = .26, p < .01), as well as the cultural variables of community connectedness (r = .19, p < .01), community standing (r = .15, p < .01), and traditional language comprehension (r = .19, p < .01). In a multivariate regression model, age, community connectedness, and community standing remained significantly associated with traditional diet. These findings may inform the design and evaluation of community-based, culturally-relevant dietary initiatives for heart health.

## Introduction

The preparation, sharing, and eating of traditional foods celebrates and sustains culture [1]. For Western Alaska Native men and women of the Norton Sound region, traditional foods provide another benefit–being high in healthy marine sources of omega-3 polyunsaturated fats, such as oil from seals, walrus, and whales, as well as salmon and other fish [2]. This region has a population of approximately 9,500 between the largest town of Nome (population 3,600) and 15 communities with populations ranging from 150 to 900 residents [3, 4]. Approximately 76% of the population is of Alaska Native heritage, primarily Inupiaq, Central Yup’ik and Siberian Yup’ik [5]. The traditional Alaska Native diet consumed in this region has been associated with many positive health outcomes, such as improved lipid profiles, better glucose tolerance, and lower levels of obesity [6–8]. Further, previous studies have found that Alaska Native people who eat more traditional foods consumed significantly more vitamin A, vitamin D, vitamin E, Iron, and omega-3 fatty acids than those with largely nontraditional diets [9, 10].

However, as processed foods have become more prevalent in rural Alaska Native communities, consumption of these traditional foods by Alaska Native people has declined over time [11, 12]. The decrease in healthy-fat rich traditional foods has corresponded with an increase in simple carbohydrate consumption, obesity, and chronic disease [13]. While cardiovascular disease differs by region in Alaska, in aggregate it is responsible for nearly 1 in 5 deaths for Alaska Native men and nearly 1 in 4 deaths for Alaska Native women, and represents the greatest cause of death in Norton Sound [14]. Further, while cardiovascular disease mortality has declined in the U.S. overall, the rate of decline is less among Alaska Native communities [15, 16]. It has been postulated that perceived stress was a contributor to markers of cardiovascular disease (obesity, high blood pressure, and high cholesterol) in American Indian and Alaska Native communities, but recent research found no such association–suggesting the role of other lifestyle factors like diet as a potential mediator [17].

Federally-funded public health programs have targeted health disparities among at-risk communities such as the remote Alaska Native communities in the Norton Sound Region; however, relatively few efforts have incorporated community-based methods that emphasize traditional foods [18]. Beyond the health-promoting effects of many traditional Alaska Native foods, recent studies have highlighted the cultural and community benefits traditional eating may provide [19]. Prior research among indigenous communities has found that traditional eating increases feelings of connectedness to one’s culture and community, which in turn has been associated with improved quality of life and greater mental well-being [20–22]. Hence, traditional eating may play a broader role in overall health than currently reflected in dietary guidelines and community-based dietary initiatives.

The current study aimed to describe and identify correlates of traditional eating practices among Alaska Native adults in the Norton Sound region of Alaska, as well as test associations between traditional eating and cultural factors such as community connectedness, community standing, and traditional language comprehension. This investigation may underscore the importance of traditional eating among Alaska Native adults in the Norton Sound Region for not only the health benefits, but also cultural and community benefits–which may lead to culturally-relevant public health programming and a generalizable framework for other communities across the country.

## Methods

### Sample

Participants were Alaska Native adults from the Norton Sound region of Alaska, recruited between 2015–2018 for a randomized clinical trial of multiple risk behavior change interventions for cardiovascular disease prevention (clinical trial registration number: NCT02137902). The Norton Sound region consists of 16 communities with population sizes ranging from approximately 120 to 3800 residents [3, 4]. Participants were recruited through comprehensive, community outreach and community engagement including three forms of media (radio, print, social), tabling in high traffic community settings, and word-of-mouth. Inclusion criteria included: Alaska Native heritage; English literacy; age 19 years or older; residing in the Norton Sound region; currently smoking 5 or more cigarettes per day; with high blood pressure (systolic/diastolic BP ≥ 140 mmHg/90 mmHg) or high cholesterol (LDL ≥ 160) or currently prescribed antihypertensives or cholesterol lowering medication [5]. Individuals who were pregnant, currently in a tobacco cessation program, taking smoking cessation medications, or had a body mass index (BMI) >50 were excluded.

### Measures

Diet was measured using a novel 34-item food frequency questionnaire (FFQ) (S1 File), which was adapted from a validated, previously designed measure used in Alaska Native communities in the Southeast Region of Alaska and developed with oversight from community stakeholders [23–25]. The FFQ was administered at the baseline interview to assess how many times in the previous week participants consumed each food item as part of a meal or snack. Response options were coded as: “Did not eat it this week” (0), “Once this week” (1), “2–3 times this week” (2.5), “4–6 times this week” (5), “Once or twice each day” (10.5), or “More than twice each day” (14). Of interest in the current analysis was the “traditional foods index”, calculated to represent the proportion of participants’ reported diet that was made up of traditional foods. The traditional foods index was calculated by dividing the number of times that traditional foods were eaten per week by total food consumed as reported on the FFQ. Two of the 34 FFQ food items were excluded from the index because their classification made them culturally ambiguous: “ice cream” could refer to nontraditional store-bought brands as well as akutaq (often referred to as Alaska Native ice cream) and “donuts”, which was indistinguishable between western or Alaska Native varieties. Of the remaining 32 items, 9 traditional food items were common to the local region and consumed by Alaska Native communities prior to western influences, such as wild berries, moose, whale (including whale fat / whale oil) and fish (including baked and fried salmon, fish and walrus soup) [26]. Similar foods were grouped to reduce the data, such as “baked salmon” and “fish soup” into “fish”, or “sodas” and “sweetened fruit drinks” into “sweet drinks”. Fruits and vegetables that were categorized as nontraditional included: apples, bananas, other whole fresh fruit, celery, canned beets, carrots, and lettuce. This resulted in 5 categories for traditional foods and 6 categories for nontraditional foods.

Community connectedness was measured with the question, “How connected do you feel to the larger community? On a scale from 1 to 10, with 1 representing the lowest amount of connectedness and 10 representing the greatest amount of connectedness.” Community standing was measured with a pictorial 10-rung ladder and the question, “People define community in different ways; please define it in whatever way is most meaningful to you. At the TOP of the ladder are the people who have the highest standing in their community. At the BOTTOM are the people who have the lowest standing in their community. Where would you place yourself on this ladder? Please draw an X where you think you stand at this time in your life, relative to other people in your community?”. Both measures were adapted from the MacArthur Scale, originally designed to measure subjective social status [27]. Traditional language comprehension was measured with the questions: “On a scale of 0–100 (0 being not at all, 100 being completely) how well do you understand your traditional language?” and recoded from a scale of 100 to a scale of 10. Prior research has found that those who have a higher connection to a language feel more connection with that language’s culture [28].

Participants reported their age and gender, and the timing of participants’ baseline assessments were coded for season with May-September categorized as “summer” and October-April as “winter” (broad cutoffs were determined with consultation of individuals familiar with the region). Lastly, participants’ community size was dichotomized to compare Nome (population approximately 3800) to smaller Norton Sound communities (populations <1000).

### Analyses

Of 299 participants enrolled in the trial and surveyed at baseline, three participants were excluded from analyses due to missing data on > 6 nutrition items and five participants were excluded for being outliers based on their total scores on the FFQ or their “traditional foods index” score (i.e., >3 standard deviations from the mean). Five participants with only one item of dietary data missing had their missing response imputed with the sample mean, rounding to the nearest integer, and included in analysis.

Independent sample t-tests were used to compare means on the traditional foods index by season (summer vs. winter), community size (~3800 residents vs. <1000 residents), and gender (men vs. women). Associations between the traditional foods index with age, community connectedness, community standing, and traditional language comprehension were measured with Spearman’s correlations. Lastly, hierarchical multiple regression was run to test associations with the traditional foods index entering all variables with significant univariate associations into the model.

This study was approved and informed consent obtained through institutional review boards at Stanford University; the University of California, San Francisco; and the Alaska Area; the Alaska Native Tribal Health Consortium Board and its manuscript and proposal review committee; and the Norton Sound Health Corporation Board of Directors and its Research Ethics Review Board. Additional information regarding the ethical, cultural, and scientific considerations specific to inclusivity in research is included in the S2 File.

## Results

### Sample description

The sample was 291 Alaska Native adults (51% men, 49% women), with a mean age of 47 years (SD = 14 years, range: 19 to 80). About a fifth (22%) of the sample was recruited from Nome and 78% from the surrounding communities in Norton Sound. By season, 48% of the sample completed the baseline survey in the summer and 52% in the winter (Table 1). Alaska Native heritage was self-identified as 59% Inupiaq, 31% Yupik and 10% multiple or another Alaska Native heritage. Biometric testing indicated 80% had hypertension and 39% had high cholesterol. Traditional language comprehension averaged a score of 5.2 (SD = 3.6, range 0 to 10). Community connectedness averaged a score of 5.7 (SD = 2.6, range 1 to 10), while community standing averaged a score of 5.3 (SD = 2.1, range 1 to 10).

**Table 1:** Characteristics of participants (n = 291).

Characteristics	mean (SD^a^) or n (%)
Age in years, mean (SD)	47 (14)
Male, n (%)	147 (51%)
Female, n (%)	144 (49%)
Location, n (%)	
Nome (~3000 residents)	65 (22%)
Other Community (<1000 residents)	226 (78%)
Surveyed by season^b^, n (%)	
Summer (May-September)	140 (48%)
Winter (October-April)	151 (52%)
Alaska Native heritage (self-identified), n (%)	
Inupiaq	173 (59%)
Yupik	89 (31%)
Multiple or Another	29 (10%)
Hypertension, n (%)	234 (80%)
High cholesterol, n (%)	113 (39%)
Traditional language comprehension^c^, mean (SD)	5.2 (3.6)
Community connectedness^c^, mean (SD)	5.7 (2.6)
Community standing^c^, mean (SD)	5.3 (2.1)

^a^SD = standard deviation

^b^Seasons represent broad cutoffs determined with consultation of individuals familiar with the region.

^c^Self report with a scale from 1–10, with 1 being low and 10 being high.

### Dietary profiles

**Fig 1:**
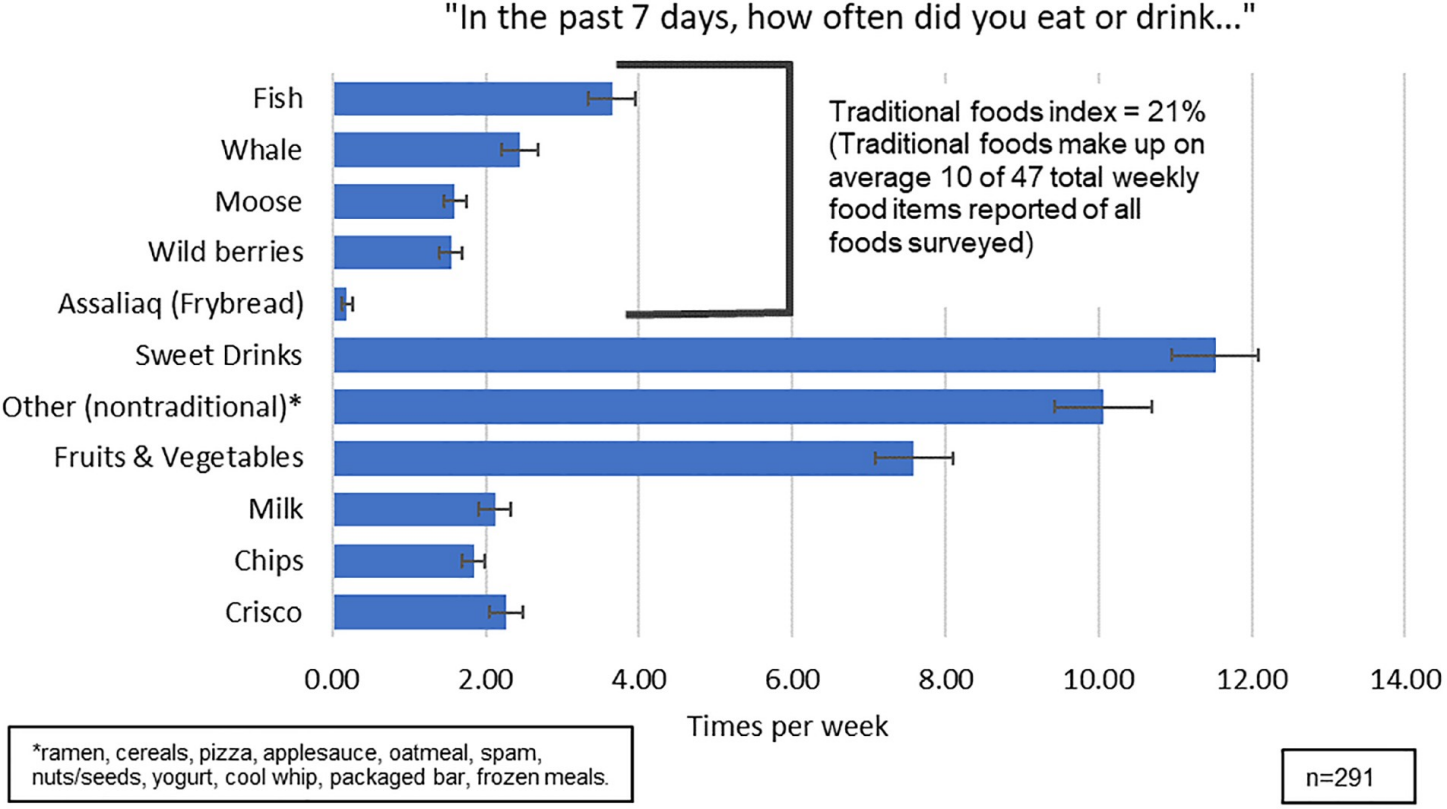
Participants’ most frequent foods consumed, shown as times per week and grouped as traditional and nontraditional.

Fig 1 shows the sample’s mean FFQ reports per week for the five traditional food groupings (top of the figure) and the six nontraditional food groups (bottom of the figure). Among the traditional foods, fish was consumed the most frequently at an average of approximately 4 times per week, while whale, moose, and wild berries each were consumed at an average of approximately 2 times per week. Assaliaq (frybread) was consumed very infrequently. For nontraditional foods, sweet drinks were consumed the most commonly at an average of approximately 12 times per week; followed by “other” (10 times per week); nontraditional fruits and vegetables (8 times per week); and milk, chips, and Crisco (2 times per week). On average, traditional eating made up 10/47 total foods reported per week, equating to a traditional foods index of 21%–or approximately 1/5^th^ of reported diet consisting of traditional foods.

### Associations with traditional food intake

Independent sample t-tests revealed significant differences in the traditional foods index for participants assessed in the summer months compared to winter months, for participants residing in smaller communities compared to Nome, and for participants identifying as men compared to women (Table 2).

**Table 2:** Traditional eating by season, community size, and gender.

		Traditional Foods Index		
Groups	N	Mean	Standard deviation	P-value
Summer (May-September)	140	23%	17%	0.03
Winter (October-April)	151	19%	15%	
Nome (~3000 residents)	66	17%	14%	0.03
Other (<1000 residents)	225	22%	17%	
Men	147	19%	16%	<0.05
Women	144	22%	16%	

Spearman correlations were significant for the associations between the traditional foods index and age (r = .26, p<0.01), community connectedness (r = .19, p<0.01), community standing (r = .15, p = 0.01), and traditional language comprehension (r = .19, p<0.01).

Hierarchical multiple regression models were run with the traditional foods index as the dependent variable. Community connectedness and community standing were tested in separate models because they were highly correlated with each other (r = 0.49) but of unique interest. Covariates were age, gender, season, community size, and traditional language comprehension. Tables 3 and 4 show the results. Age and community connectedness, as well as age and community standing remained significant predictors of participants’ traditional diet. The total percent of variance accounted for in both full models was 10%.

**Table 3:** Hierarchical multiple regression model predicting the traditional food index with community connectedness.

Variable	b	SE	B	t	P-value
Age	0.17	0.07	0.15	2.30	0.02
Season (summer vs. winter)	-3.19	1.90	-.010	-1.68	0.09
Community size (other vs. Nome)	-2.92	2.32	-.08	-1.26	0.21
Gender (men vs. women)	1.96	1.91	0.60	1.02	0.31
Traditional language comprehension	0.04	0.03	0.91	1.45	0.15
Community connectedness	0.86	0.38	0.14	2.26	0.02

**Full model** F (6, 267) = 5.17, p < .01, R^2^ = 0.10 (adj-R^2^ = 0.08)

**Table 4:** Hierarchical multiple regression model predicting the traditional food index with community standing.

Variable	b	SE	B	t	P-value
Age	0.18	0.08	0.15	2.36	0.02
Season (summer vs. winter)	-3.29	1.94	-0.10	-1.70	0.09
Community size (other vs. Nome)	-3.66	2.35	-0.09	-1.56	0.12
Gender (men vs. women)	2.03	1.95	0.62	1.04	0.30
Traditional language comprehension	0.05	0.03	0.10	1.63	0.11
Community standing	1.04	0.47	0.13	2.21	0.03

**Full model** F(6, 268) = 5.01, p < .01, R^2^ = 0.10 (adj-R^2^ = 0.08)

## Discussion

In a sample of Alaska Native men and women from the Norton Sound region with multiple risk factors for cardiovascular disease, traditional dietary patterns were associated with perceived community standing and community connectedness; and the association held after controlling for age, gender, season, community size, and traditional language comprehension. While causality cannot be determined from these cross-sectional data, it can be concluded that those who reported eating more traditional foods also reported greater connection and standing in their community–factors that promote health and well-being [12, 29–34].

Research on cultural and community connectedness and health behaviors in Indigenous adults has shown positive association for physical activity in multiple cross-sectional studies [31–33]. One study with African American adults found cultural identification associated with increased leisure-time physical activity, healthier diet, and less smoking [29]. Research with the Nebraskan Omaha Tribe reported associations between culture and diabetic control, while a study with the Navajo Nation found greater ethnic identity associated with better oral health [30, 34]. More relevant to the current study, past research with Alaska Native adults has found traditional food use associated with engagement in traditional physical activity and other cultural behaviors [12]. In the context of prior research, the current findings provide support that traditional eating may play an important role in community and cultural connectedness and its related health benefits.

Other findings of interest include greater traditional eating in the summer months, among women than men, among older participants, and among participants residing in smaller communities as opposed to Nome. More traditional eating in the summer months is consistent with subsistence activities. Food items such as wild berries, moose, and fish are more easily and readily available in Alaska in the summer [35]. However, it is important to consider that weather variations and subsistence patterns in the Norton Sound region are much more complex than just summer and winter as dichotomized in this study, and the months selected to categorize them (May-September for summer and October-April for winter) are imperfect. While cross-sectional and longitudinal research has found greater traditional food consumption among Alaska Native women compared to men, in the present study gender differences were no longer significant when controlling for age [36]. The greater influence of age on traditional dietary behaviors is to be expected and has been previously described [37]. Younger generations likely encounter more Western influences, and previous studies have found globalization and climate change have led to a decrease in subsistence activities among younger Alaska Native people [38].

The finding that those in smaller communities ate more traditionally than those residing in Nome is consistent with the observed greater availability of Westernized, processed foods at a lower cost in Nome. Affordability is a likely mediator given prior research findings that increased food costs in rural, primarily Indigenous communities contribute to food insecurity and increased consumption of processed foods [39]. However, other confounders may complicate investigations into the relationship between community size and traditional eating such as Nome serving as the hub of the region and being culturally and demographically distinct from the other more remote communities in Norton Sound.

Fish being the traditional food item consumed most frequently (approximately 4 times per week) followed by whale, moose, and wild berries (approximately 2 times per week) is as expected, given the coastal region’s close proximity to the ocean. These findings are similar to other studies of traditional food consumption among Alaska Native people, performed across multiple regions [12, 36]. It should be noted that sweet drinks were consumed at a high frequency in the sample at approximately 11 times per week, consistent with prior research in the region [40]. Interventions that promote drinking water to reduce sweet drinks have been effective in other communities [41]. Important to consider, however, is the accessibility of clean, running water in rural Alaska Native communities; those lacking potable tap water may consume more sweet drinks [42]. Future research should assess access to both traditional foods and potable water sources in the region, as the value placed in this research is dependent on traditional foods being accessible and inexpensive.

This study had several important limitations, such as the inherent complexity of assessing dietary intake along with a novel FFQ and traditional eating index. There is a tradeoff faced between adding more FFQ food items to better capture diet versus response fatigue from too many options available–for this reason there was a limited selection of traditional foods included. The FFQ was administered one time and relied on recall from the previous week. With nutrition assessments, people tend to overreport desirable foods and underreport undesirable foods [43]. The present study adds another layer to this desirability bias, as traditional foods may be considered more desirable to report. In addition, the “traditional foods index” reflected the frequency that foods were eaten, rather than caloric intake. A sweetened drink differs in calories to that of moose, for example. Further, the majority of the traditional food items could be thought of as the main course of a meal (e.g., moose, fish), while the majority of nontraditional foods were drinks or snacks. This could skew one’s dietary index as less traditional than it actually may be, as drinks and snacks may outnumber meals during the course of a week. However, our finding of an average traditional foods index of 21% corresponds with similar, caloric based studies among Alaska Native communities, which have found that traditional foods account for approximately 22% of overall energy intake, and which similarly varied by age, geographic location, as well as educational level [9]. This close agreement provides support, but the traditional food index should be further validated before major conclusions are drawn.

An additional limitation is that English literacy was required for study participation. One person was excluded for this reason. In the context of health disparities research and to maximize inclusivity, future research ought to adapt measures to interview and engage interpreter services. Of the three primary Alaska Native heritage groups represented in our study, Inupiaq, Central Yup’ik and Siberian Yup’ik, varying degrees of Native language use has been reported. While Native language use in the communities of Norton Sound, and specifically among people without English literacy, is unknown, across the entire state of Alaska about 25% of Inupiaq adults speak a dialect of Inupiaq; while 48% of Central Yup’ik adults and 90% of Siberian Yup’ik speak a dialect of Yup’ik [44].

### Implications

In rural Alaska Native communities and many other diverse communities around the world, traditional eating can be a healthy alternative to increasingly abundant, highly processed, and unhealthy food items. This study introduces a novel measure of traditional food consumption and illustrates that traditional eating is associated with social and cultural benefits, namely a greater feeling of connectedness to one’s community and a higher level of perceived community standing. As public health programs design and deliver initiatives in chronic disease prevention and food assistance to at-risk communities, the importance of incorporating traditional foods must be considered. Rather than broad, one-size-fits-all models, leadership should engage with stakeholders via community based participatory research to find ways to promote and increase traditional food availability, consumption, and celebration. In addition to potentially improving health, promotion of traditional diets may yield valued community-related social benefits.

## Supporting information

S1 File Food frequency questionnaire (FFQ). (PDF)

S2 File Checklist for global inclusivity of research. (DOCX)

## References

[1] Kittler PG, Sucher KP, Nelms M. Food and Culture. 2nd ed. Boston, MA: Cengage Learning; 2017. 1–23 p.

[2] Johnson J, Nobmann ED, Asay E. Factors related to fruit, vegetable and traditional food consumption which may affect health among Alaska Native People in Western Alaska. Int J Circumpolar Health. 2012;71. doi: 10.3402/ijch.v71i0.17345 22456043 PMC3417710

[3] Norton Sound Health Corporation. Promoting Healthy Generations: Fiscal Year 2016 Annual Report. [Internet]. 2016. https://www.nortonsoundhealth.org/wp-content/uploads/FY16-Annual-Report.pdf

[4] U.S. Census Bureau. QuickFacts: Nome Census Area, Alaska [Internet]. 2010. https://www.census.gov/quickfacts/fact/table/nomecensusareaalaska,AK/PST045217

[5] Prochaska JJ, Epperson A, Skan J, Oppezzo M, Barnett P, Delucchi K, et al. The Healing and Empowering Alaskan Lives Toward Healthy-Hearts (HEALTHH) Project: Study protocol for a randomized controlled trial of an intervention for tobacco use and other cardiovascular risk behaviors for Alaska Native People. Contemp Clin Trials. 2018 Aug;71:40–6. doi: 10.1016/j.cct.2018.06.003 29864548 PMC6636857

[6] Annuzzi G, Rivellese AA, Wang H, Patti L, Vaccaro O, Riccardi G, et al. Lipoprotein subfractions and dietary intake of n-3 fatty acid: The genetics of coronary artery disease in alaska natives study. Am J Clin Nutr. 2012. doi: 10.3945/ajcn.111.023887 22572646 PMC3349453

[7] Adler AI, Boyko EJ, Schraer CD, Murphy NJ. Lower Prevalence of Impaired Glucose Tolerance and Diabetes Associated With Daily Seal Oil or Salmon Consumption among Alaska Natives. Diabetes Care. 1994 Dec;17(12):1498–501. doi: 10.2337/diacare.17.12.1498 7882827

[8] Bersamin A, Luick BR, King IB, Stern JS, Zidenberg-Cherr S. Westernizing Diets Influence Fat Intake, Red Blood Cell Fatty Acid Composition, and Health in Remote Alaskan Native Communities in the Center for Alaska Native Health Study. J Am Diet Assoc. 2008 Feb;108(2):266–73. doi: 10.1016/j.jada.2007.10.046 18237575 PMC6542563

[9] Bersamin A, Zidenberg-Cherr S, Stern JS, Luick BR. Nutrient intakes are associated with adherence to a traditional diet among Yup’ik Eskimos living in remote Alaska Native communities: the CANHR Study. Int J Circumpolar Health. 2007;66(1):62–70. doi: 10.3402/ijch.v66i1.18228 17451135

[10] Fohner AE, Wang Z, Yracheta J, O’Brien DM, Hopkins SE, Black J, et al. Genetics, diet, and season are associated with serum 25-hydroxycholecalciferol concentration in a Yup’ik study population from southwestern Alaska. J Nutr. 2016. 10.3945/jn.115.223388 PMC4725435 26661839

[11] O’Brien DM, Thummel KE, Bulkow LR, Wang Z, Corbin B, Klejka J, et al. Declines in traditional marine food intake and vitamin D levels from the 1960s to present in young Alaska Native women. Public Health Nutr [Internet]. 2017 Jul 1 [cited 2021 Aug 20];20(10):1738. Available from: /pmc/articles/PMC5274583/ doi: 10.1017/S1368980016001853 27465921 PMC5274583

[12] Redwood DG, Ferucci ED, Schumacher MC, Johnson JS, Lanier AP, Helzer LJ, et al. Traditional foods and physical activity patterns and associations with cultural factors in a diverse Alaska Native population. Int J Circumpolar Health. 2008 Sep;67(4):335–48. doi: 10.3402/ijch.v67i4.18346 19024803 PMC2925499

[13] Ryman TK, Boyer BB, Hopkins S, Philip J, Beresford SAA, Thompson B, et al. Associations between diet and cardiometabolic risk among Yup’ik Alaska Native people using food frequency questionnaire dietary patterns. Nutr Metab Cardiovasc Dis. 2015;25(12):1140–5. doi: 10.1016/j.numecd.2015.08.003 26607703 PMC4684467

[14] Howard B V, Metzger JS, Koller KR, Jolly SE, Asay ED, Wang H, et al. Alaska Native People: Western Alaska Tribal Collaborative for Health (WATCH). Am J Public Health. 2014;104(7):1334–41. 24754623 10.2105/AJPH.2013.301614 PMC4056205

[15] Howard B V., Devereux RD, Cole SA, Davidson M, Dyke B, Ebbesson SOE, et al. A genetic and epidemiologic study of cardiovascular disease in Alaska natives (GOCADAN): design and methods. Int J Circumpolar Health. 2005 Jul;64(3):206–21. doi: 10.3402/ijch.v64i3.17985 16050315

[16] Jolly SE, Howard B V., Umans JG. Cardiovascular Disease Among Alaska Native Peoples. Curr Cardiovasc Risk Rep. 2013 Dec;7(6):438–45. doi: 10.1007/s12170-013-0362-5 24367710 PMC3869403

[17] Nikolaus CJ, Sinclair K, Buchwald D, Suchy-Dicey AM. Association of stress and resilience with cardiometabolic health among American Indian and Alaska Native adults. Prev Med Reports [Internet]. 2021 Dec 1 [cited 2021 Aug 20];24:101517. Available from: https://linkinghub.elsevier.com/retrieve/pii/S2211335521002072 doi: 10.1016/j.pmedr.2021.101517 34458080 PMC8379486

[18] Satterfield D, DeBruyn L, Santos M, Alonso L, Frank M. Health Promotion and Diabetes Prevention in American Indian and Alaska Native Communities—Traditional Foods Project, 2008–2014. MMWR Suppl. 2016. doi: 10.15585/mmwr.su6501a3 26916637

[19] Blanchet R, Batal M, Johnson-Down L, Johnson S, Louie C, Terbasket E, et al. An Indigenous food sovereignty initiative is positively associated with well-being and cultural connectedness in a survey of Syilx Okanagan adults in British Columbia, Canada. BMC Public Health. 2021;21(1405). 10.1186/s12889-021-11229-2 PMC8283975 34271895

[20] Bersamin A, Izumi BT, Nu J, O’brien DM, Paschall M. Strengthening adolescents’ connection to their traditional food system improves diet quality in remote Alaska Native communities: results from the Neqa Elicarvigmun Pilot Study. Transl Behav Med [Internet]. 2019 Oct 1 [cited 2021 Aug 19];9(5):952–61. Available from: https://academic.oup.com/tbm/article/9/5/952/5579392 doi: 10.1093/tbm/ibz087 31570921 PMC6937549

[21] Henson M, Sabo S, Trujillo A, Teufel-Shone N. Identifying Protective Factors to Promote Health in American Indian and Alaska Native Adolescents: A Literature Review. J Prim Prev [Internet]. 2017 Apr 1 [cited 2021 Aug 19];38(1–2):5. Available from: /pmc/articles/PMC5313316/ doi: 10.1007/s10935-016-0455-2 27826690 PMC5313316

[22] Utsey SO, Chae MH, Brown CF, Kelly D. Effect of ethnic group membership on ethnic identity, race-related stress, and quality of life. Cult Divers Ethn Minor Psychol. 2002 Nov;8(4):366–77. 10.1037/1099-9809.8.4.367 12416322

[23] Slattery ML, Murtaugh MA, Schumacher MC, Johnson J, Edwards S, Edwards R, et al. Development, Implementation, and Evaluation of a Computerized Self-Administered Diet History Questionnaire for Use in Studies of American Indian and Alaskan Native People. J Am Diet Assoc. 2008 Jan;108(1):101–9. doi: 10.1016/j.jada.2007.10.008 18155994 PMC2474673

[24] Kolahdooz F, Simeon D, Ferguson G, Sharma S. Development of a quantitative food frequency questionnaire for use among the Yup’ik people of Western Alaska. PLoS One. 2014. doi: 10.1371/journal.pone.0100412 24963718 PMC4070930

[25] Johnson JS, Nobmann ED, Asay E, Lanier AP. Developing a validated alaska Native food frequency questionnaire for western Alaska, 2002–2006. Int J Circumpolar Health. 2009. doi: 10.3402/ijch.v68i2.18319 19517870

[26] Muller MK. Promoting or Protecting Traditional Knowledges? Tensions in the Resurgence of Indigenous Food Practices on Vancouver Island. Int Indig Policy J. 2018;9(4).

[27] Brown RA, Adler NE, Worthman CM, Copeland WE, Costello EJ, Angold A. Cultural and community determinants of subjective social status among Cherokee and White youth. Ethn Heal. 2008. doi: 10.1080/13557850701837302 18701990 PMC4075651

[28] Oster RT, Grier A, Lightning R, Mayan MJ, Toth EL. Cultural continuity, traditional Indigenous language, and diabetes in Alberta First Nations: A mixed methods study. Int J Equity Health. 2014. 10.1186/s12939-014-0092-4 PMC4210509 25326227

[29] Airhihenbuwa CO, Kumanyika SK, TenHave TR, Morssink CB. Cultural identity and health lifestyles among African Americans: A new direction for health intervention research? Ethn Dis. 2000. 10892821

[30] Penn-Kennedy J, Barber C. Cultural Identity and Control of Diabetes among Members of the Omaha Tribe in Nebraska. Wicazo Sa Rev. 1995.

[31] Henderson KA, Ainsworth BE. Sociocultural perspectives on physical activity in the lives of older african american and american indian women: A cross cultural activity participation study. Women Heal. 2000. doi: 10.1300/J013v31n01_01 11005217

[32] Duncan GE, McDougall CL, Dansie E, Garroutte E, Buchwald D, Henderson JA. Association of American Indian cultural identity with physical activity. Ethn Dis. 2014. 24620441 PMC3970840

[33] Thompson JL, Wolfe VK, Wilson N, Pardilla MN, Perez G. Personal, social, and environmental correlates of physical activity in Native American women. Am J Prev Med. 2003. 10.1016/s0749-3797(03)00165-x 14499810

[34] Brega AG, Henderson WG, Harper MM, Thomas JF, Manson SM, Batliner TS, et al. Association of ethnic identity with oral health knowledge, attitudes, behavior, and outcomes on the Navajo nation. J Health Care Poor Underserved. 2019. 10.1353/hpu.2019.0013 PMC6400317 30827975

[35] Ryman TK, Boyer BB, Hopkins S, Philip J, O’brien D, Thummel K, et al. Characterising the reproducibility and reliability of dietary patterns among Yup’ik Alaska Native people. Br J Nutr. 2015. doi: 10.1017/S0007114514003596 25656871 PMC4621269

[36] Redwood DG, Day GM, Beans JA, Hiratsuka VY, Nash SH, Howard B V, et al. Alaska Native Traditional Food and Harvesting Activity Patterns over 10 Years of Follow-Up. Curr Dev Nutr. 2019. doi: 10.1093/cdn/nzz114 31723724 PMC6834783

[37] Nobmann ED, Ponce R, Mattil C, Devereux R, Dyke B, Ebbesson SOE, et al. Dietary intakes vary with age among Eskimo adults of Northwest Alaska in the GOCADAN study, 2000–2003. J Nutr. 2005. doi: 10.1093/jn/135.4.856 15795447

[38] Walch A, Bersamin A, Loring P, Johnson R, Tholl M. A scoping review of traditional food security in Alaska. International Journal of Circumpolar Health. 2018. doi: 10.1080/22423982.2017.1419678 29292675 PMC5757232

[39] Stotz S, Brega AG, Henderson JN, Lockhart S, Moore K. Food Insecurity and Associated Challenges to Healthy Eating Among American Indians and Alaska Natives With Type 2 Diabetes: Multiple Stakeholder Perspectives. J Aging Health. 2021. doi: 10.1177/08982643211013232 34167350 PMC8647808

[40] Ballew C, Ross Tzilkowski A, Hamrick K, Nobmann ED. The Contribution of Subsistence Foods to the Total Diet of Alaska Natives in 13 Rural Communities. Ecol Food Nutr. 2006 Jan;45(1):1–26.

[41] Kenney EL, Gortmaker SL, Carter JE, Howe MCW, Reiner JF, Cradock AL. Grab a cup, fill it up! an intervention to promote the 1 of drinking water and increase student water consumption during school lunch. Am J Public Health. 2015. 10.2105/AJPH.2015.302645 PMC4539814 26180950

[42] Wojcicki JM, Young MB, Perham-Hester KA, De Schweinitz P, Gessner BD. Risk factors for obesity at age 3 in Alaskan children, including the role of beverage consumption: Results from Alaska PRAMS 2005–2006 and Its three-year follow-up survey, CUBS, 2008–2009. PLoS ONE. 2015. doi: 10.1371/journal.pone.0118711 25793411 PMC4368660

[43] Miller TM, Abdel-Maksoud MF, Crane LA, Marcus AC, Byers TE. Effects of social approval bias on self-reported fruit and vegetable consumption: a randomized controlled trial. Nutr J. 2008 Dec;7(1):18. doi: 10.1186/1475-2891-7-18 18588696 PMC2467430

[44] Alaska Native Languages [Internet]. Fairbanks, Alaska; 2021. https://www.uaf.edu/anlc/languages/siberianyupik.php

